# Improvement in Pharmacological Activity of Mahkota Dewa (*Phaleria macrocarpa* (Schef. Boerl)) Seed Extracts in Nanoemulsion Dosage Form: *In vitro* and *In vivo* Studies

**DOI:** 10.2174/2211738511666230602100045

**Published:** 2023-10-05

**Authors:** Eriska Agustin, Muhammad Insanu, Rachmat Mauludin

**Affiliations:** 1 School of Pharmacy, Bandung Institute of Technology, Bandung, 40132, Indonesia

**Keywords:** Mahkota Dewa seed, nanoemulsion, anti-oxidant, anti-inflammatory, inflammation, plethysmometer method

## Abstract

***Background*:** Mahkota Dewa (*Phaleria macrocarpa*) seed has various phytochemical compounds and low pharmacological activities, including antioxidant and anti-inflammatory activities.

***Objective*:** This research aimed to study nanoemulsion preparations of Mahkota Dewa seed (NE-BMD) for their anti-oxidant and anti-inflammatory properties.

***Methods*:** The nanoemulsion was prepared using an ultrasonication probe and followed by selecting two formulations, F7 and F8. The anti-oxidant activity test was carried out using the DPPH method, meanwhile, the anti-inflammatory activity test was conducted using the protein denaturation method with Bovine Serum Albumin (BSA) for *in vitro* studies. In addition, for *in vivo* studies, the plethysmometer method was used with 1% carrageenan as an inducer.

***Results*:** The characterization of NE-BMD preparations showed that the particle size and polydispersity index were 26,83 ± 1,27 nm (PI: 0.36 ± 0.03) and 30.73 ± 1.50 nm (PI: 0.32 ± 0.06) for NE-BMD F7 and F8 formulation, respectively. In addition, the anti-oxidant activity test revealed that the IC_50_ values of NE_BMD F7 and F8 were 15.62 ± 1.40 µg/ml and 28.39 ± 4.69 µg/ml, respectively. The protein denaturation test showed that the IC_50_ values for NE-BMD F7 and F8 were 94.39 ± 1.24 µg/ml and 196.63 ± 1.61 µg/ml, respectively. Meanwhile, the study of anti-inflammatory *in vivo* for NE-BMD F7 with a 1 g/kg BW dose showed a significant improvement in anti-inflammatory activity compared to BMD extract.

***Conclusion*:** This research suggests that due to the smaller drug particle size, the nanoemulsion dosage form of Mahkota Dewa seed extract has anti-oxidant and anti-inflammatory activities, thus emerging as an adjunct alternative treatment for inflammation.

## 1 INTRODUCTION

Mahkota Dewa is an Indonesian plant from the Thymelaceae family that grows in tropical areas. Empirically, Mahkota Dewa is believed to have a potential pharmacological activity to treat hypertension, diabetes, cancer, and diuretics. Natural phytochemicals from Mahkota Dewa are also reported to have biological activities, including anti-oxidant, anti-bacterial, anti-hypertensive, anti-hyperglycemic, and anti-inflammatory activities [1]. The results of the phytochemical test showed the presence of secondary metabolites, including flavonoids, saponin glycosides, phenolic compounds, steroids, tannins, and terpenoids in the Mahkota Dewa seed extract. The main components, such as flavonoids, saponin glycosides, phenolic compounds, steroids,tannins, and terpenoids, are present in the Mahkota Dewa seed, which contributes to its pharmacological activity [2]. Currently, natural anti-oxidants derived from plants are widely used because they have pharmacological potential and low side effects.

Flavonoids are the main class of polyphenolic compounds and are widely found in fruits, leaves, flowers, and seeds of Mahkota Dewa. According to a previous study [1], Mahkota Dewa fruit skin extract had an anti-oxidant activity with an IC_50_ value of 71.97%, while the lowest was reported for the seed extract, *i.e*., only 54.44% at 300 µg/ml concentration. These results indicate the presence of low anti-oxidant activity in the Mahkota Dewa seed extract.

The ability of Mahkota Dewa seed as an anti-inflammatory agent may be attributed to the presence of phenolic and flavonoid compounds [3]. The anti-inflammatory activity of Mahkota Dewa fruit extract, induced by lipopolysaccharide/Interferon alfa, demonstrated a nitric oxide inhibition value of 63.4 ± 2.70% -69.5 ± 1.40% [4]. Flavonoids are capable of inhibiting nitric oxide production and iNOS expression but their strength depends on the structure of the flavonoid class [5].

Flavonoid compounds have polar and non-polar properties, which affect the bioavailability of the drug in the body. The development of the new drug delivery system can increase the solubility of the non-polar flavonoid properties, which, in turn, is expected to increase the pharmacological activity, especially for oral route administration [6].

Nanoemulsions are colloidal particulate systems with sizes ranging from 10 to 1000 nm. Nanoemulsion carriers are solid spheres and their surface is amorphous and lipophilic. This thermodynamically unstable system can be stabilized by using an emulsifying agent (emulsifier). Nanoemulsion contains oil, emulsifying agent, and an aqueous phase. An emulsifying agent can impart stability to reduce the surface tension between oil and the aqueous phase. The small size of the nanoemulsion makes it transparent [7]. Notably, the absence of creaming and maintenance of appearance, color, odor, and consistency characterizes a stable nanoemulsion [8]. In comparison with emulsion, stable nanoemulsion requires higher concentrations of emulsifying agent.

The increased anti-inflammatory activity of Boswellia serrata extract, made of nanoemulsion, was reported to increase the influx by 3.25 times *ex vivo* and increased its anti-inflammatory activity compared to its nanosuspension dosage form [9]. In addition, the nanoemulsion of sweet orange peel plant extract (*Citrus sinensis* (L.) Osbeck) increased the Area Under Curve (AUC) and C_max_ values by 5.67 and 2.64 times, respectively, compared to nanosuspension extract [10]. It can be concluded that the nanoemulsion dosage form can increase the pharmacological activity. Therefore, nanoemulsion provides many advantages in drug delivery systems [11].

Several previous studies regarding the activity test of BMD still have low pharmacological activities. Therefore, the purpose of this research is to develop the nanoemulsion dosage form from ethyl acetate of Mahkota Dewa seed extract, which is expected to increase several pharmacological activities, including anti-oxidant and anti-inflammatory activities in *in vitro* and *in vivo* studies.

## 2 MATERIALS AND METHODS

### 2.1 Materials

The following materials were used in this study: ethyl acetate (Brataco), Cremophor^®^ RH40 (BASF, Germany), polyethylene glycol 400 (PEG-400, Brataco), oleic acid (Brataco), ethanol p.a (Merck, Germany), methanol p.a. (Merck, Germany), quercetin (Sigma Aldrich, MERCK), 2.2-diphenyl-1-picrylhydrazil (DPPH, Sigma Aldrich, MERCK), ascorbic acid (Sigma Aldrich, MERCK), Bovine Serum Albumin Type A9647 (Sigma Aldrich, MERCK), tyrosinase enzyme (Sigma Aldrich, MERCK), L-DOPA (Sigma Aldrich, MERCK), sodium diclofenac (Dexa Medica), alumunium chloride, sodium acetate, buffer phosphate pH 6.8, dimethyl sulfoxide, Tris Buffer Saline (TBS), hydrochloric acid, Dragendorrf’s reagent, Lieberman-Bouchard reagent, Mayer reagent, Stiasny reagent, amyl alcohol, magnesium powder, ferric chloride (FeCl_2_), chloroform, ether, acetic acid anhydrous, acetic acid glacial, ammonia, sodium hydroxide, carrageenan, gelatin, and aquadeion.

### 2.2 Methods

#### 2.2.1 Preparation of Mahkota Dewa Seed (BMD) Extract

Mahkota Dewa seeds were obtained from the garden in West Bandung and determined at Herbarium Bandungense, School of Life Science and Technology, Bandung Institute of Technology. The BMD extract was made by maceration method using ethyl acetate solvent for 3 x 24 hours with ratios of 1:10 for the first day and 1:5 for the second and third days. The process of obtaining the liquid extract was carried out by the solvent evaporation process using a rotary evaporator, which was optimized by using a water bath to obtain a thick extract.

#### 2.2.2 Determination of Total Flavonoid Content

The test procedure was carried out by pipetting 0.5 ml of the standard solution of quercetin and each comparison sample solution separately and adding 1.5 ml of ethanol p.a., 0.1 ml of 10% aluminium chloride, 0.1 ml of sodium acetate 1 M, and 2.8 ml of water. The mixture was shaken and let stand for 30 minutes at room temperature, and then the absorption of the standard solution and each comparison sample solution was measured at a wavelength of 415 nm. The blank was measured in the same way without adding aluminium chloride. A calibration curve for the absorbance was created and the concentration of the sample solution was calculated [12].

#### 2.2.3 Determination of Total Phenolic Content

The test procedure was carried out by pipetting 1 ml of each comparison sample solution and the standard solution of gallic acid separately and adding 5.0 ml of diluted Folin-Ciocalteu LO (75% in water). The mixture was shaken and let stand for 8 minutes. After that, 4.0 ml of 1% NaOH was added, and then the mixture was incubated for 1 hour. The absorption of the standard solution and each comparison sample solution was measured at a wavelength of 730 nm. The blank was measured in the same way without adding the sample solution. The calibration curve was measured and the concentration of the sample solution was calculated [12].

#### 2.2.4 Preparation of Nanoemulsion BMD Extract

The nanoemulsion BMD extract dosage form was made by the method that has been developed previously [13], namely the modified sonication method. The oil phase used BMD extract and oleic acid as oil, Cremophor^®^ RH 40 as a surfactant, and PEG-400 as a co-surfactant with optimization of concentration ratio. The mixing was carried out in stages by mixing the oil phase and Cremophor^®^ RH40, and then those phases were stirred using a magnetic stirrer at 400 rpm for 15 minutes. Subsequently, PEG 400 was added as a co-surfactant to the mixture, and then the mixture was stirred using a magnetic stirrer at 400 rpm for 90 minutes. After that, the particle size reduction of the mixture was carried out with the bath-type sonicator for 45 minutes.

The nanoemulsion BMD was added with aquadeion until 100% and then it was stirred at a low speed using a magnetic stirrer until they were mixed and nanoemulsion BMD was formed. The final nanoemulsion consisted of 3% of BMD extract, meanwhile, F7 and F8 were selected for further study.

#### 2.2.5 Characterization of Nanoemulsion BMD

The characterization of nanoemulsion BMD dosage form included the determination of particle size, polydispersity index, and zeta potential using the Delsa^TM^ Nano Zeta Potential and Submicron Particle Size Analyzer (Beckman Coulter). Particle morphology was tested using Transmission Electron Microscope (JEOL JEM 1400). Meanwhile, the determination of transmittance was carried out using UV-Visible Spectrophotometer.

#### 2.2.6 Antioxidant Activity Testing

The determination of the antioxidant activity of BMD extract and nanoemulsion BMD using the DPPH method was modified [14]. The determination of antioxidant activity was carried out using DPPH as the reagent, sample solution with various concentrations, and standard solution using ascorbic acid at a ratio of 1:1, which was incubated at 25°C for 30 minutes in a dark place. The absorbance was measured using UV-Visible Spectrophotometer at a wavelength of 517 nm.

#### 2.2.7 Anti-inflammatory Activity Testing (In vitro)

The measurement of anti-inflammatory activity *in vitro* was carried out by pipetting 500 µl of sodium diclofenac solution, BMD extract solution, and nanoemulsion BMD solution with various concentrations, and then each solution was added with 1% BSA until 5 ml, respectively. Each solution was incubated for 30 minutes at room temperature and then heated for 5 minutes at 70-72°C using a water bath. After that, it was cooled for 25 minutes at room temperature and the absorbance was measured using UV-Visible Spectrophotometer at a wavelength of 660 nm.

#### 2.2.8 Anti-inflammatory Activity Testing (In vivo)

The measurement of anti-inflammatory activity *in vivo* was carried out based on the modification done previously [15] using the plethysmometer method, namely the edema induction method with carrageenan on the feet of rats. Before the treatment, the rats fasted for 18 hours but drinking water was still given. The rats were divided into 6 groups and each group consisted of 3 white rats and the weights were around 150-200 g. Each rat was measured with a plethysmometer as the base volume. After the initial data were obtained, each group was given a negative control, positive control, BMD extract solution, and nanoemulsion BMD solution. The rats were divided into 6 groups of 3 as follows:

Group 1 = 0.5% CMC 1 ml/200 g/kg BW

Group 2 = Sodium diclofenac solution at a dose of 40 mg/kg BW.

Group 3 = BMD extract at a dose of 0.5 g/kg BW.

Group 4 = BMD extract at a dose of 1 g/kg BW.

Group 5 = nanoemulsion BMD at a dose of 0.5 g/kg BW.

Group 6 = nanoemulsion BMD at a dose of 1 g/kg BW.

After 1 hour of administrating the test and control solution, each rat was injected with 0.1 ml of 1% carrageenan solution in the soles of its feet (subplantar). After 1 hour of carrageenan injection, the volume of the feet at the injection site was measured by a plethysmometer. The measurement was taken every hour for 6 hours after injection of carrageenan and continued until 24 hours. The volume inflammation was the difference between the volumes of the sole before and after the injection of carrageenan.

#### 2.2.9 Statistical Analysis

Statistical analysis was carried out using SPSS software version 27.

## 3 RESULTS AND DISCUSSION

### 3.1 Determination of Total Flavonoid and Total Phenolic Content

Mahkota Dewa seed was obtained by extracting with the maceration method. The maceration method was chosen because the processing technique is simple and can be used to extract thermolabile compounds or those not known to be resistant to heat. The solvent used in the maceration process was ethyl acetate. The purpose of selecting a semipolar solvent was to attract polar and non-polar compounds from the Mahkota Dewa seed. In addition, ethyl acetate solvent is a good solvent for extraction because it is volatile, not hygroscopic, and has low toxicity [16, 17]. The liquid extract was carried out by thickening process and evaporating the solvent using a rotary evaporator to obtain a concentrated extract and assist using a water bath. The yield of the extract was 35.097% and the extract was in the form of oil.

The phenolic compound is the largest group of secondary metabolites found in green plants. According to a study [1], one of the phenolic compounds, including flavonoids, has several biological activities. They act as anti-oxidants, inhibitors of enzyme hydrolysis and oxidative, and anti-inflammatory. Recent scientific studies have proved that phenolic compounds can protect cells from free radical damage. The results of the determination of total phenolic and total flavonoid contents in the Mahkota Dewa seed extract were 34.41 ± 0.06 mg Gallic Acid Equivalent (GAE)/g extract sample and 55.89 ± 3.03 mg QE/g extract sample, respectively. Quercetin was used as a standard solution for flavonoids and gallic acid as a standard solution for phenolic.

### 3.2 Characterization of Nanoemulsion BMD (NE-BMD) Dosage Form

**Fig. (1):**
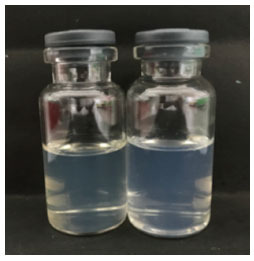
Nanoemulsion BMD extract F7 (left) and F8 (right).

The process of making nanoemulsion BMD (NE-BMD) extract used the sonication method with oil, surfactant, and co-surfactant components in a certain ratio. The optimization results of the concentration of each component containing 1% oleic acid (F7) and no oleic acid (F8) were chosen. The results of the globule size measurement showed a transparent and brownish liquid (Fig. **1**). Nanoemulsions BMD F7 and F8 have the criteria for nanoparticle preparation with a particle size of less than 100 nm and polydispersity index value of below 0.5, indicating that nanoemulsions BMD F7 and F8 have the homogeneous dispersion system (Table **1**) with better stability.

**Fig. (2):**
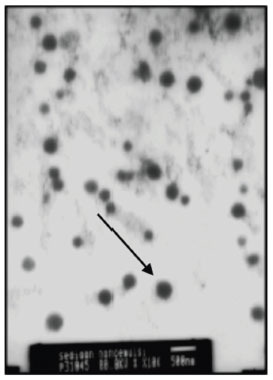
The globule morphology of the NE-BMD F7.

The results of particle morphology analysis using TEM showed a spherical shape with a magnification of 10,000 times and a resolution of 200 nm (Fig. **2**). It was found that the oil globules in the nanoemulsion BMD were distributed uniformly. The transmittances NE-BMD F7 and F8 were determined to be 92.31% and 94.62%, respectively. Nanoemulsion BMD can be assumed to have a transparent color like water.

The low potential zeta in this study is assumed by the use of a large amount of nonionic surfactant (Cremophor^®^ RH40) and the presence of co-surfactant PEG 400. The negative value of the zeta potential may occur due to the ionization of the components forming the interfacial layer surrounding the nanoemulsion globules (surfactant and co-surfactant) [18]. Surfactant Cremophor^®^ RH40 is a non-ionic surfactant that will form steric barriers to maintain the physical stability of the preparations.

**Fig. (3):**
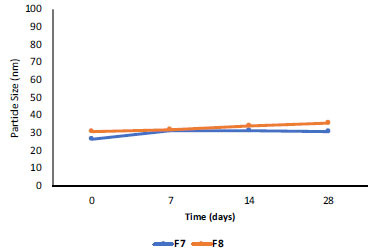
Particle size stability of nanoemulsion BMD F7 and F8 at room temperature (25°C).

**Fig. (4):**
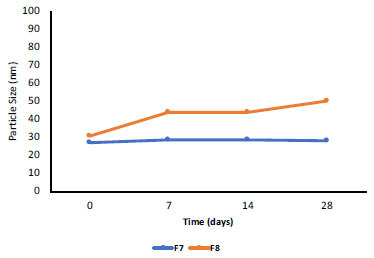
Particle size stability of nanoemulsion BMD F7 and F8 at 4°C temperature.

**Fig. (5):**
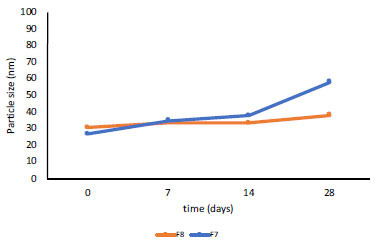
Particle size stability of nanoemulsion BMD F7 and F8 at 40°C temperature.

The results of the stability testing at 3 different temperatures showed a slight increase in the globule sizes, which tended to be stable. The globule size of NE-BMD F7 increased at 4°C and tended to be stable at 25°C and 40°C (Fig. **3**), while the globule size of NE-BMD F8 increased at 25°C and tended to be stable at 4°C and 40°C (Figs. **4** and **5**). Temperature and duration of storage can affect emulsion viscosity and aggregation formation because oil phase crystallization induces partial incorporation of emulsion droplets or leads to conformational changes of surfactant molecules [19]. Based on the previous research [20], the changes in globule size at storage temperature can provide varying viscosity of emulsion preparations, which produce a destabilizing effect.

The changes in emulsion viscosity will affect the mass transport kinetics of surfactants and oil molecules at the boundary between the oil and water phases. It can affect the formation of oil droplets and induces variations in globule size, potential zeta, and turbidity. In addition, temperature affects the deposition rate of the interfacial film by changing the adsorption rate, interfacial characteristics, and compressibility of the film by changing the solubility of the surfactant in the emulsion phase. The two preparations are still considered stable (the sizes obtained are under 100 nm) [21].

### 3.3 Antioxidant Activity Testing

Oxygen is an important chemical element in the metabolism of aerobic organisms, but it can trigger unwanted reactions and can form free radicals. These free radicals are a side product of normal cell production and if they increase significantly, they can result in damage to molecules, such as protein, lipid, RNA, and DNA, and can increase the risk of diseases, such as cancer, diabetes, and premature aging. Antioxidants can protect cells with various mechanisms, one of which is the conversion of ROS (Reactive Oxygen Species) into non-radical species [22].

The determination of antioxidant activity is based on the value of Inhibitory Concentration (IC_50_), which indicates that the sample concentration can inhibit 50% of free radical scavenging. The results of antioxidant activities of NE-BMD F7 and F8 showed a significant decrease in IC_50_ value compared to BMD extract. Antioxidant activities of NE-BMD F7 and F8, BMD extract, and the standard solution are presented in Table **2** and Fig. (**6**). The nanoemulsion preparation was able to increase the antioxidant activity 13 times (15.62 ± 1.40 µg/ml) for NE- BMD F7 and 7 times (28.39 ± 4.69 µg/ml) for NE-BMD F8 compared to BMD extract (207.01 ± 23.02 µg/ml). According to the literature, the seed extract was only 54.44% at 300 µg/ml concentration. These results indicated the presence of low antioxidant activity in the *Phaleria macrocarpa* seed extract (1). The antioxidant activity was improved by NE-BMD. It is considered that the antioxidant activity of phenolic compounds from Mahkota Dewa seed is due to their high redox potential, allowing them to act as reducing agents, hydrogen donors, and singlet oxygen quenchers [3]. In addition, the antioxidant activity is determined by their structure, in particular the electron delocalization over an aromatic nucleus. An increase in the antioxidant activity in NE-BMD could be caused by the solubility of extract into small globules at the nanometer range. So, the surface area will increase and accelerate contact with free radicals, which makes NE-BMD able to generate free radicals from DPPH [22].

### 3.4 Anti-inflammatory Activity Testing (*In vitro*)

Inflammation is an immune response of the body due to tissue damage. Inflammation can trigger complex reactions that usually cause pain. One of the therapies commonly used is anti-inflammatory Non-Steroidal Anti-Inflammatory Drugs (NSAIDs). However, the use of NSAIDs is limited because of their side effects on the digestive tract. Mahkota Dewa seed has anti-inflammatory activity because of flavonoid compounds [23]. In this research, for the determination of anti-inflammatory activity, we used the protein denaturation inhibition method with BSA.

**Fig. (7):**
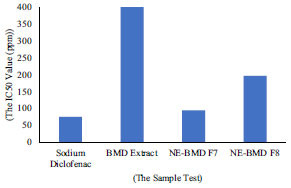
The IC_50_ value of anti-inflammatory activity *in vitro*.

The results of this research about the anti-inflammatory activity of NE-BMD showed that the IC_50_ values of the NE-BMD F7 and F8 were 94.39 ± 1.24 µg/ml and 196.63 ± 1.61 µg/ml compared to that of BMD extract of 401.46 ± 1.01 µg/ml (Table **3**). Based on these data, the potential to inhibit protein denaturation of NE-BMD decreased the IC_50_ value compared to BMD extract (Fig. **7**).

The results of this experiment are supported by a previous study [24], which stated that flavonoids inhibit the lipoxygenase pathway directly in inflammation. It causes inhibition of eicosanoid biosynthesis and inactivates free radicals that can attract various inflammatory mediators.

Protein denaturation is one of the causes of inflammation, where the state of losing protein structure and function goes through several triggers, such as temperature, pH, pressure, electricity, chemical mixtures, and reducing agents [25].

The increase of anti-inflammatory activity NE-BMD due to the globule size in the nanometer range causes an increase in the amount of BMD extract globule in the preparations. One of the causes of the inflammatory process is an increase in temperature, which causes protein denaturation so that the protein composition becomes unstable and causes the formation of free radicals. The NE-BMD preparation has a small globule size, large surface area, and relatively homogeneous globule distribution that can cause an increase in interaction or bond between molecules in BSA and BMD extract in nanoemulsion preparation. It can inhibit protein denaturation and the formation of free radicals that stimulate the release of inflammatory mediators [4].

### 3.5 Anti-inflammatory Activity Testing (*In vivo*)

The *in vivo* testing of anti-inflammatory activity was a follow-up test after the *in vitro* test using the BSA protein denaturation method. The nanoemulsion BMD F7 preparation was chosen as the *in vivo* sample test because it had the highest anti-inflammatory activity based on BSA protein denaturation testing.

The composition of NE-BMD F7 contained 1% oleic acid, which was assumed to help increase the anti-inflammatory activity, followed by antioxidant activity. The anti-inflammatory activity was determined by inducing 1% carrageenan in male Wistar rats because it could cause acute inflammatory symptoms and a relatively short swelling time of 2-3 hours. The test method is the Winter method using a plethysmometer. The principle of the plethysmometer is based on Archimedes’s law, in which the applied pressure equals the released pressure. The interpretation of the results of this test is the percentage value of the inhibition of inflammation from samples [15].

**Fig. (8):**
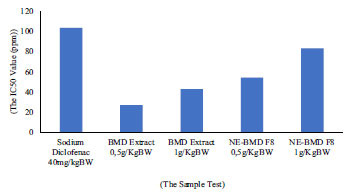
Total percentage of the inhibition of anti-inflammatory activity *in vivo*.

The determination of anti-inflammatory activity was carried out on negative control 0.5% Na-CMC, positive control 40 mg/kgBW sodium diclofenac, BMD extract, and NE-BMD preparations with doses of 0.5 g/kgBW and 1 g/kgBW, respectively. The negative control Na-CMC, where the highest swelling or percentage of inflammation occurred, appearing at the 3^rd^ hour, was 89.69 ± 9.65% and it was a significant and statistical difference with % of inflammation from the sample test solution (*P* > 0.05). The phase of histamine and serotonin released and increased the prostaglandin at the 3^rd^ hour. The percentage value of inhibition at the 3^rd^ hour for sodium diclofenac as a positive control was 85.13 ± 3.02%, and BMD extracts were 41.72 ± 13.92% and 52.09 ± 5.39% at doses of 0.5 g/kgBW and 1 g/kgBW, respectively. The % values of inhibition of inflammation from NE-BMD F7 preparations were 62.76 ± 4.17% and 66.54 ± 3.52% at doses of 0.5 g/kgBW and 1 g/kgBW, respectively (Fig. **8**). The NE-BMD F7 had a continuous inhibition with similar % value of inhibition of inflammation from the 3^rd^ to 6^th^ hours at the dose of 1 g/kgBW. There was no inhibitory effect of the BMD extract, positive control, or NE-BMD preparation.

The NE-BMD preparation containing oil extract can also increase the stability of the extract encapsulated in nanoemulsion preparation from gastric degradation and produce an extended-release formulation. Nanoemulsion preparations have the ability to increase membrane diffusion slowly (sustained and released) [26].

The increase in the anti-inflammatory activity of NE-BMD preparation can also be due to small particle size and homogeneous particle distribution, so it is assumed to increase cellular uptake of the active compound molecules from the extract by passive transport through the stomach wall and increase the drug absorption rate [27].

**Fig. (9):**
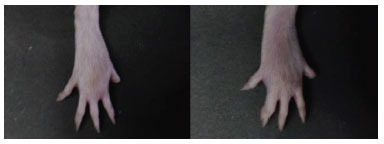
The feet of rats after being injected with 1% carrageenan at 0 hour (Right) and after being injected by NE-BMD 1 g/kgBW dose (Left) at the 3^rd^ hour.

The presence of anti-inflammatory activity in the BMD extract can be due to the presence of flavonoids in the extract. Flavonoids are known to be able to inhibit the activity of phospholipase enzymes so that they can inhibit the formation of arachidonic acid as well as prostaglandins, thromboxanes, prostacyclins, and leukotrienes, which are responsible for the inflammatory process [28]. In addition, flavonoids are able to inhibit nitric oxide production, iNOS expression, and leukocyte accumulation in inflammatory areas [29]. In statistical testing, there was a significant difference between BMD extract at a dose of 0.5 g/kgBW and NE-BMD preparation at doses of 0.5 g/kgBW and 1 g/kgBW at the 3^rd^ hour (*p* < 0.05). However, there was no significant difference (*p* > 0.05) between the NE-BMD 0.5 g/kgBW and NE-BMD 1 g/kgBW. From the results of the anti-inflammatory activity test, it can be concluded that the NE-BMD preparations can exhibit an anti-inflammatory effect (Fig. **9**).

## CONCLUSION

Mahkota Dewa seed extract can be formulated in nanoemulsion dosage form (NE-BMD). The NE-BMD preparations can contribute to increasing several pharmacological activities *in vitro* and *in vivo,* including anti-oxidant and anti-inflammatory. The NE-BMD can be considered an additional alternative treatment for inflammation. However, this study needs further research to prove the isolation compounds, other pharmacological activities, and their effects of toxicity.

## Figures and Tables

**Fig. (6):**
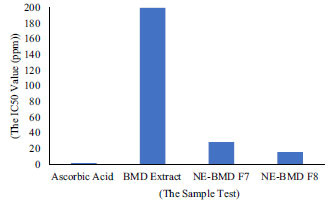
The IC_50_ value of antioxidant activity testing.

**Table 1:** Physical characteristics of NE-BMD preparation.

**The Sample**	**Particle Size (nm)**	**Polydispersity Index**	**Potential Zeta (mV)**
NE-BMD F7	26.83 ± 1.27	0.36 ± 0.03	-5.87
NE-BMD F8	30.73 ± 1.50	0.32 ± 0.06	-6.10

**Note:** Data represent mean ± SD, n = 3.

**Table 2:** The result of the anti-oxidant activity testing.

**The Sample Test**	**The IC_50_ Value (μg/mL)**
Ascorbic acid (positive control)	1.62 ± 0.04
BMD extract	207.01 ± 23.02
NE-BMD F7	15.62 ± 1.40
NE-BMD F8	28.39 ± 4.69

**Note:** Data represent mean ± SD, n = 3. Means with the same alphabet along the column are not significantly different (*p* < 0.05) by one-way ANOVA.

**Table 3:** The result of the anti-inflammatory testing *in vitro.*

**The Sample Test**	**The IC_50_ Value (μg/mL)**
Sodium diclofenac (positive control)	7.,93 ± 3.53
BMD extract	401.46 ± 1.01
NE-BMD F7	94.39 ± 1.24
NE-BMD F8	196.63 ± 1.61

**Note:** Data represent mean ± SD, n = 3. Means with the same alphabet along the column are not significantly different (*p* < 0.05) by one-way ANOVA.

## Data Availability

The data that support the findings of this study are available from the corresponding author (RM) upon request.

## LIST OF ABBREVIATIONS

AUC: Area under Curve
GAE: Gallic Acid Equivalent
NSAIDs: Non-Steroidal Anti-Inflammatory Drugs
